# Eco-Friendly Biocomposites from Chestnut Waste: Production, Optimization, Characterization, and Application

**DOI:** 10.3390/polym17050616

**Published:** 2025-02-25

**Authors:** Simão B. Silva, Olga M. Freitas, Elsa F. Vieira, Amália Gomes, Ana R. Carreiras, Diogo C. Moreira, Púria Esfandiari, João F. Silva, Cristina Delerue-Matos, Valentina F. Domingues

**Affiliations:** 1 REQUIMTE/LAQV, ISEP, Polytechnic of Porto, Rua Dr. António Bernardino de Almeida 431, 4249-015 Porto, Portugal; 1181156@isep.ipp.pt (S.B.S.); omf@isep.ipp.pt (O.M.F.); emfva@isep.ipp.pt (E.F.V.); cmm@isep.ipp.pt (C.D.-M.); 2 CICECO—Aveiro Institute of Materials, Department of Chemistry, University of Aveiro, 3810-193 Aveiro, Portugal; amalia.gomes@ua.pt; 3 M4S—Materials for Sustainability, ISEP, Polytechnic of Porto, 4249-015 Porto, Portugal; arz@isep.ipp.pt (A.R.C.); 1181755@isep.ipp.pt (D.C.M.); pur@isep.ipp.pt (P.E.); jfs@isep.ipp.pt (J.F.S.)

**Keywords:** chestnut-based biocomposites, response surface methodology, mechanical properties, water absorption, thermal properties, sustainability

## Abstract

This study explores the valorization of non-commercial chestnut waste from the Portuguese chestnut industry to develop biocomposites. The composites were obtained by hot compression molding, and a Box–Behnken Design model was employed to optimize the mechanical, thermal, and water resistance properties of the chestnut-based composite, using fruit and shell fibers, respectively, as the polymeric matrix and reinforcement agent. The optimal formulation, comprising 70% chestnut, no glycerol, a molding temperature of 120 °C, and applying a pressure of 2.93 MPa for 30 min, achieved a Flexural Strength of 9.00 MPa and a Flexural Modulus of 950 MPa. To enhance water resistance, shellac was added as a natural hydrophobic coating. Water interaction tests indicated that shellac-treated biocomposites exhibited superior water resistance, absorbing approximately two times less water than those containing glycerol or untreated samples. Thermal analysis revealed that glycerol acted as a plasticizer, improving flexibility and reducing the glass transition temperature. Additionally, the chestnut-based biocomposite demonstrated an out-of-plane thermal conductivity of 0.79 W/m·K, categorizing it as a thermal insulator. The final prototype application was a candle holder, showcasing the potential for the practical and sustainable use of chestnut-based composite. This research highlights the potential for chestnut waste to be repurposed into eco-friendly products, offering an alternative to conventional plastics and contributing to a circular economy.

## 1. Introduction

Plastic pollution is a global environmental crisis, with significant amounts of waste infiltrating ecosystems and contributing to widespread ecological harm [1]. In response, efforts have been directed toward developing sustainable and biodegradable alternatives to petroleum-based plastics. Among these alternatives, biocomposites have emerged as a promising solution. Biocomposites are materials composed of natural fibers or particles embedded in a biopolymer matrix [2]. This combination leverages the biodegradability of biopolymers and the enhanced mechanical and thermal properties provided by natural reinforcements, making them suitable for several applications. Natural reinforcements can be sourced from plant-based materials such as flax; hemp; and sisal and agricultural residues like rice husk, sugarcane bagasse, and coconut husk [3]. Utilizing these materials improves the performance of biocomposites and reduces carbon footprint by relying on renewable and sustainable resources.

The European chestnut (*Castanea sativa* Mill.) has experienced a significant increase in production due to growing consumer awareness of the chestnut’s nutritional value. Indeed, global chestnut production has more than doubled from 945,990 tons in 2001 to 2,131,240 tons in 2022 [4]. Consumption varies considerably, with Europeans consuming approximately 0.5 kg of chestnuts per year compared to 2.5 kg per person in China, Japan, and Korea, while Americans consume only about 0.05 kg annually [5]. Although chestnuts are highly valued, their processing generates substantial waste, including shells, burs, and non-commercial chestnuts. These by-products are rich in starch [6], lignin [7], and cellulose [8], with the fibers in the shells and burs serving as valuable reinforcement to enhance the mechanical and thermal properties of biocomposites, which presents a promising opportunity for sustainable reuse. In Portugal, the chestnut industry is heavily concentrated in the Trás-os-Montes region, which accounts for over 80% of the country’s chestnut production [9]. Despite its economic importance, it is estimated that between 40 and 50% of chestnuts are lost during production due to factors such as infestation, rot, and processing waste. This amounts to approximately 10,850 tons of waste annually, valued at around EUR 21.7 million [10]. Disposal methods for chestnut by-products, such as the pericarp (outer shell) and tegument (inner shell), often involve their use as a heat source or their return to the soil [11], but these practices can lead to greenhouse gas emissions and soil degradation. Given the need for sustainable solutions, the valorization of chestnut by-products represents a viable approach to reducing waste and enhancing the value of this natural resource [12]. A comprehensive review article has addressed chestnut-based composite production, exploring various production methods, processing parameters, and recycling techniques [13], including burs [14,15,16,17], shells [18,19,20,21,22], and wood flour [23], employing diverse methods such as compression molding, extrusion, hand lay-up, and chemical grafting. Composites reinforced with chestnut burs typically demonstrate superior water barrier properties, with water absorption rates between 0.54% [14] and 1.60% [15]. In contrast, chestnut-shell-reinforced composites generally show enhanced mechanical properties, with tensile strengths between 11 MPa [19] and 48 MPa [20]. It is noteworthy that these outcomes are influenced by the different matrix systems and production methods across studies. To date, no comparative study has systematically evaluated the performance of chestnut shells versus burs within a consistent composite production framework.

The goal of this work is valorizing Portuguese chestnut industry residues while ensuring a fully natural and sustainable material. This study investigates the use of non-commercial chestnuts—specifically, those from harvests that fail to meet commercial standards—to produce biocomposites. These biocomposites, fabricated through hot compression molding, consist solely of chestnut-derived residues, with kernel starch as the polymeric matrix, reinforced with fibers from the inner shells. Unlike previous studies, which rely on external matrix systems such as urea-formaldehyde resins [14], polyester [17,18], or polyhydroxyalkanoates (PHAs) [19], this work pioneers a fully chestnut-based biocomposite formulation, integrating glycerol as a plasticizer. The research focuses on optimizing the composition (including chestnut and glycerol content) and pressing temperature, as well as evaluating the biocomposites’ performance in terms of mechanical, thermal, and water-resistant properties. The addition of shellac as a natural hydrophobic coating further enhances their water-resistant properties. A significant novelty of this research is the development of a practical application, demonstrated through the fabrication of a prototype candle holder, showcasing the potential of chestnut waste as a sustainable and functional material. However, further studies are required to assess the economic viability and environmental sustainability of scaling up this process for industrial applications.

## 2. Materials and Methods

**Figure 1:**
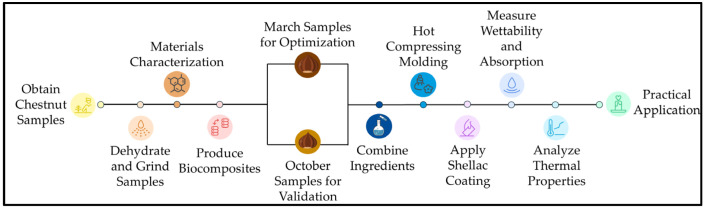
Schematic representation of the workflow for chestnut-based biocomposite development.

Figure 1 illustrates the step-by-step process involved in the development and characterization of chestnut-based biocomposites, from raw material acquisition to practical application.

### 2.1. Material

Non-commercial chestnuts (Longal variety) were provided by Sortegel (Bragança, Portugal) in two batches: March 2024 and October 2024. Chestnut samples refer to chestnuts unsuitable for sale due to size (<20 mm), defects (no kernel), and rottenness. These samples have a percentage amount of shells due to the small size of the fruit. Among other components, the sample contains the following: proteins < 11.22 g/100 g Dry Weight (DW) [24], starch <53.6 g/100 g DW [10], cellulose <29.2 g/100 DW [8], and Klason Lignin <47.0 [25] Both batch samples were dehydrated at 41 °C for 24 h using an Excalibur 3926TB 9-Tray Electric Food Dehydrator (Sacramento, CA, USA) and stored with silica gel to maintain low moisture. Before composite production, they were ground into 2 mm particles using a Black Coffee Grinder 200 W Natural 30 RRK from Aigostar (Fuzhou, China). The March chestnut sample was used to optimize the mechanical properties and characterize the composites. The October chestnut sample, with higher fruit content and starch proportion, was used in this BBD validation study to investigate the impact of seasonal variability on the mechanical properties of the biocomposites. Glycerol (>99% purity) from A.M.C. Cunha, Lda (Porto, Portugal) was used as a plasticizer. Shellac Q4008 (100% purity) was acquired from SPD (Gondomar, Portugal) is a long-chain polyester type of resin consisting of inter- and intra-esters of polyhydroxy carboxylic acids where some acids are aliphatic long-chain hydroxy acids, and some are sesquiterpene acids [26], served as a waterproofing coating.

### 2.2. Material Characterization

#### FTIR

Fourier Transform Infrared Spectroscopy (FTIR) analysis was performed using a Thermo Scientific Nicolet 6700 FT-IR spectrometer (Waltham, MA, USA) with an MCT/A detector. For sample preparation, 10 mg of non-commercial chestnut or shellac was finely ground and mixed with 200 mg of potassium bromide using a mortar. The mixture was then placed in an insert holder and compressed for 5 min to form a pellet. The sample was analyzed in the 750 cm^−1^ to 4000 cm^−1^ range.

### 2.3. Production and Optimization of Biocomposites

The biocomposites were produced using hot compression molding with a custom-made press from Gislotica, applying a pressure of 2.93 MPa for 30 min. Disk-shaped specimens with a diameter of 160 mm were created (see Supplementary Materials, Figure S1) allowing for easy division into multiple samples for mechanical testing. The pressed material consisted of non-commercial chestnut waste, combining chestnut shells and fibers as reinforcement and kernel starch as the polymeric matrix. Glycerol was incorporated as a plasticizer to enhance flexibility. Response Surface Methodology (RSM) with a Box–Behnken Design (BBD) was used to investigate the influence of the independent variables, which comprised chestnut content (X1, 65–85%), glycerol content (X2, 0–30%), and molding temperature (X3, 80–120 °C), on the mechanical properties’ Flexural Strength (Y1, MPa) and Flexural Modulus (Y2, MPa). Preliminary studies were conducted using a univariate approach to ensure that the potential maximum and minimum points of the RSM were identified. These tests were based on a composite density of 2.3 g/cm^3^ (considered 100% composition), but for RSM optimization, the density was not fixed, allowing the model to determine both the optimal composition and corresponding density. Table 1 shows the design matrix for the 17 experimental runs (each run was performed in triplicate) while the Supplementary Materials present the coded and actual levels of the independent variables (see Supplementary Material, Table S1). The BBD results were analyzed using Design Expert software version 13.0.5.0 with ANOVA for statistical significance and regression analysis for model adequacy. Variables with *p* > 0.05 were excluded for model refinement. The optimal response values (Y) were identified by solving the regression equations and examining the response surface and contour plots generated from the predictive equations of RSM. To verify the model’s accuracy, experiments were carried out using optimized critical values and chestnut samples from two batches: March 2024 and October 2024. Desirability indices were used to determine the ideal conditions for maximizing the Flexural Strength and Flexural Modulus biocomposite properties.

### 2.4. Shellac Gum Preparation and Application

To improve water resistance, a shellac-based coating was applied only after biocomposite production. Shellac gum was prepared by dissolving in alcohol (>96% purity) at a 1:10 (g:mL) ratio, stirring until dissolved, and left to cure for two days [27]. The solution was applied evenly with a brush to selected specimens (wettability and water absorption essays).

### 2.5. Biocomposite Characterization

#### 2.5.1. Mechanical Properties

Flexural tests were conducted following ISO 14125 standards [28] using a SHIMADZU AG-I 10 KN (Kyoto, Japan). Testing was performed at a crosshead speed of 2 mm/min and 80 mm distance between supports. Samples with 120 mm of length and 15 mm of width with an average of 6 mm of thickness were tested until rupture. Three specimens were tested for each sample to obtain reliable data on the Flexural Strength and Flexural Modulus, which were calculated from the slope of the linear portion of the Flexural Strength–displacement curve.

#### 2.5.2. Morphology

Scanning Electron Microscopy (SEM) was used to observe the morphology of the optimized ground biocomposite. Samples were fixed on support stubs, previously covered with carbon tape and gold-coated, for the evaluation of surface morphology using Quanta 400FEG ESEM/EDAX Genesis X4M equipment from FEI (Hillsboro, OR, USA).

#### 2.5.3. Wettability

Wettability was measured by placing a 3 μL droplet of distilled water on the composite surface and capturing the contact angle using a goniometer OCA-20 apparatus from DataPhysics Instruments (Filderstadt, Germany), equipped with a high-resolution camera and analysis software SCA20 M4 version 6.1. To ensure accuracy, ten measurements per sample were averaged, with results recorded immediately at room temperature. Both untreated and shellac-treated biocomposites (composed of 75% chestnut at 120 °C) were analyzed to evaluate the effect of shellac as a hydrophobic coating.

#### 2.5.4. Water Absorption

Water absorption tests were performed according to the ASTM D570 standard [29]. Samples (50 × 50 × 5 mm) were immersed in distilled water for 2, 24, and 96 h; wiped to remove the surface water; and weighed on an analytical MS105 balance with an accuracy of ±0.0001 g from Mettler Toledo (Greifensee, Switzerland). The tests included specimens with 75% chestnut, 75% chestnut with shellac, and 75% chestnut with 30% glycerol, all produced at 120 °C. The percentage weight increase was calculated to assess the impact of shellac and glycerol using Equation (1):

Increase in Weight (%) = (Wet weight − Conditioned weight)/(Conditioned weight) × 100(1)

#### 2.5.5. Thermal Conductivity

Thermal conductivity (out-of-plane) was measured using a custom-made device tested at 48 °C for accuracy with standardized materials. An 8 mm thick composite sample was placed inside an insulated box with internal resistances and thermocouples to monitor temperature on both sides. The sample tested contained 75% chestnut and 30% glycerol at 120 °C.

#### 2.5.6. TGA

Thermogravimetric analysis was performed using an STA 443 F3 Jupiter system (Netzsch, Gebrüder-Netzsch-Straße, Selb, Germany) under an air atmosphere (50 mL/min). Approximately 10 mg of powdered composite was heated from 25 to 800 °C at a heating rate of 20 °C/min. Derivative thermogravimetry (DTG) curves were derived from TGA data and processed using Netzsch Proteus Thermal Analysis standard software version 6.1. Runs 4 and 5 were selected as representative formulations to assess the influence of glycerol, as they contain the same 75% chestnut content, with 0% glycerol in run 4 and 30% glycerol in run 5, allowing a direct comparison of the plasticizer effect on thermal behavior. A temperature of 80 °C was chosen for a broader analysis across a wider temperature range.

#### 2.5.7. DSC

DSC analysis was conducted using the same STA 443 F3 Jupiter (Netzsch) system, with samples heated from 25 to 350 °C at a rate of 2 °C/min under an air atmosphere (50 mL/min). Glass transition temperature (Tg) was determined using the tangent method, following the same sample selection criteria as for TGA to evaluate the effect of glycerol content on thermal properties.

### 2.6. Candle Holder Application

A candle holder was selected as a practical application for the optimized biocomposite due to its straightforward design and minimal strength requirements. Production was carried out using a custom-built pilot-scale press (see Supplementary Materials, Figure S1), equipped with a heating system powered by six 220 W resistances, a water-cooling system, manual pressure controls, and precise temperature regulation via integrated sensors and a digital display [30]. Aluminum molds were used to shape the candle holder’s design (see Supplementary Materials, Figure S1).

## 3. Results and Discussion

### 3.1. Characterization by FTIR

The FTIR spectra of shellac and non-commercial chestnut (see Supplementary Materials, Figure S2) highlight distinct functional groups characteristic of their compositions. Shellac exhibits a broad O–H stretching band at 3452 cm^−1^, indicative of hydroxyl-rich compounds such as aleuritic, shellolic, and jalaric acids [31]. The peaks of 2923 cm^−1^ and 2857 cm^−1^ correspond to the C–H stretching vibrations of aliphatic chains, probably from aleuritic acid. A strong band at 1723 cm^−1^ confirms the presence of C=O stretching, associated with carboxyl functional groups, typical of shellac resins. Identical spectra were obtained in other study [32]. Non-commercial chestnuts show a similar O–H stretching absorption at 3429 cm^−1^. The C–H stretching peak at 2922 cm^−1^ suggests aliphatic content, while a strong band at 1652 cm^−1^ corresponds to conjugated carbonyl (C=O) groups, correlated with flavonoids [33], and lignin-derived compounds [34]. The presence of C–O stretching at 1017 cm^−1^ suggests contributions from polysaccharides (cellulose and hemicellulose) [35]. These spectral differences confirm that shellac is predominantly composed of esterified hydroxy acids, which contribute to its hydrophobic nature, making it an ideal resin for coatings and waterproofing applications. In contrast, non-commercial chestnuts are rich in polyphenols and carbohydrates, which can enhance mechanical interfacial adhesion, making them suitable as a reinforcement material in biocomposites.

### 3.2. Optimization of Mechanical Properties

**Figure 2:**
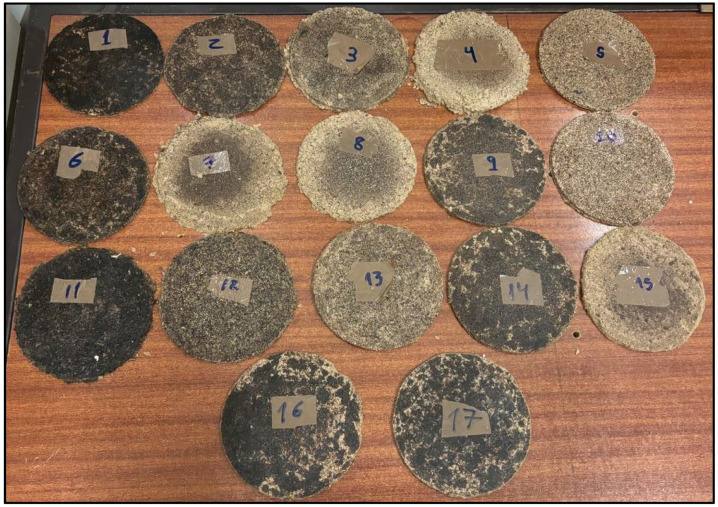
Biocomposite samples produced according to the experimental design.

The visual aspect of the developed biocomposites is illustrated in Figure 2, where a general trend can be observed: higher glycerol content results in a darker brown color. Table 1 displays the Flexural Strength and Flexural Modulus experimental and predicted values for 17 composites produced under various conditions established by a BBD.

The experimental values for Flexural Strength (Y1) ranged from 0.177 ± 0.020 MPa (Run 5) to 9.90 ± 0.14 MPa (Run 7). For the Flexural Modulus (Y2), values varied from 4.31 ± 0.65 MPa (Run 5) to 940 ± 230 MPa (Run 8). There was a reasonable agreement between the experimental and predicted values for both responses, validating the model’s predictive accuracy (see Supplementary Materials, Figure S3). The ANOVA and fit statistic results for the modified linear model of Flexural Strength are shown in the Supplementary Materials (Table S2).

ANOVA indicates that the independent variables’ glycerol content (X2) and temperature (X3) significantly impacted Flexural Strength (*p* < 0.0001). The model itself was highly significant (*p* < 0.0001), with an F-value of 43.59, confirming a strong fit. The lack of fit was not significant (*p* = 0.55), suggesting the model accurately represents the data. The model demonstrated a coefficient of determination (R^2^) of 0.8616, explaining 86.16% of the response variability, and an adjusted R^2^ of 0.8419, supporting its reliability. The predicted R^2^ of 0.7894 aligned well with the adjusted R^2^, indicating good predictive capability. The Adeq Precision value was 21.98, exceeding the threshold of 4, confirming a strong signal-to-noise ratio. The final regression Equation (2) for Flexural Strength, expressed in coded factors, is as follows:

Flexural Strength = 3.90 − 3.01X2 + 2.22X3 (2)

**Figure 3:**
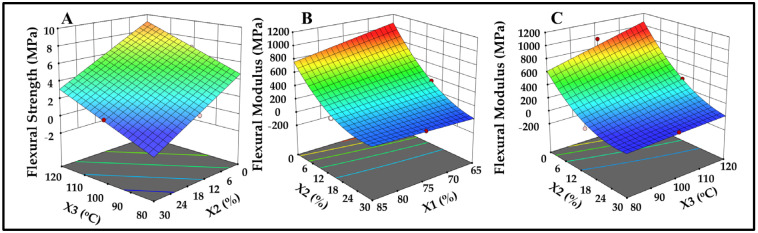
Response surface 3D plots for BBD correlations: (**A**) glycerol (X2, %) and temperature (X3, °C) for Flexural Strength (MPa); (**B**) chestnut (X1, %) against glycerol (X2, %) for Flexural Modulus (MPa); (**C**) glycerol (X2, %) against temperature (X3, °C) for Flexural Modulus (MPa).

The contour plot (Figure 3A) illustrates that Flexural Strength is highest (up to 10.0 MPa) at lower glycerol levels (0–6%) combined with higher temperatures (110–120 °C). This highlights the importance of selecting an appropriate molding temperature, which should be above the polymer melting point (256–258 °C for pure starch) but below the fiber degradation temperature (around 568 °C, as indicated by previous TGA analysis on chestnut fibers in this study) to prevent material damage [36]. Temperatures up to 120 °C were effective, as higher values led to composite burning and structural compromise. Glycerol functions as a plasticizer, reducing intermolecular forces to enhance flexibility [37,38]. However, excessive glycerol softens the matrix excessively, lowering mechanical strength, as shown by the reduced Flexural Strength at higher glycerol levels [39]. Balancing glycerol content is essential to maintain mechanical integrity and ensure a suitable trade-off between flexibility and rigidity (see Supplementary Materials, Table S2). The analysis indicates significant effects from chestnut content (X1, *p* = 0.0414), glycerol content (X2, *p* < 0.0001), and temperature (X3, *p* = 0.0006). Interaction terms X1X2 (*p* = 0.0285) and X2X3 (*p* = 0.0202) were moderately significant, with X2^2^ showing a non-linear impact (*p* = 0.0002). The model was highly significant (*p* < 0.0001) with an R^2^ of 0.9614, demonstrating robust predictive power. The final regression Equation (3) for the Flexural Modulus is as follows:

Flexural Modulus = 160.26 − 59.14X1 − 331.46X2 + 123.97X3 + 91.41X1X2 − 98.63X2X3 + 200.34X2^2^ (3)

Figure 3B shows that increased chestnut content (X1) and glycerol content (X2) reduces the Flexural Modulus. The lowest values (4.3 ± 0.7 MPa) are observed at higher glycerol percentages (30%), as glycerol significantly reduces stiffness by increasing the matrix’s flexibility (*p* < 0.0001). Chestnut fibers from chestnut shells, being softer components, also contribute to reducing stiffness and the Flexural Modulus by decreasing the overall rigidity of the composite (*p* = 0.0414) [40,41]. Figure 3C shows that the Flexural Modulus decreases at high glycerol (30%) and low temperature (80 °C), showing increased flexibility due to the plasticizing effect of glycerol. Higher temperatures with lower glycerol content result in greater stiffness of biocomposites. Glycerol degradation begins above 135 °C, so temperatures up to 120 °C maintain the structural integrity of biocomposites [42].

### 3.3. Validation of the Optimal Composition Predicted by a BBD

Experimental results closely matched the model’s predictions, confirming its accuracy. The optimal condition for Flexural Strength was found to be 75% chestnut (comprising chestnuts with <20 mm size, defects—no kernel and rottenness), 0% glycerol, and a molding temperature of 120 °C. These optimal conditions yield an experimental Flexural Strength of 9.28 ± 0.13 MPa, which is closely aligned with the predicted value of 9.14 MPa, with a desirability index of 0.913. The Flexural Modulus of the biocomposite was maximized with 67% chestnut and 0% glycerol at 118 °C; the experimental value of 970 ± 48 MPa agreed with the predicted value of 980 MPa, with a desirability index of 1.000. The combined optimal composition for both mechanical properties was 70% chestnut and 0% glycerol at 120 °C, resulting in 9.00 ± 0.40 MPa of Flexural Strength and 950 ± 11 MPa for the Flexural Modulus. Predicted values were 9.00 MPa and 10 × 10^2^ MPa, with a combined desirability of 0.945, indicating the model’s strong ability to optimize the mechanical properties of the composites.

For comparative purposes, the mechanical properties of the biocomposite produced with chestnut samples from October 2014 under identical optimized conditions (70% chestnut, 0% glycerol, 120 °C) were also evaluated. This biocomposite showed a slightly lower Flexural Strength of 7.64 ± 1.69 MPa but a higher (*p* < 0.05) Flexural Modulus of 1445 ± 175 MPa. These differences could be attributed to variations in the batch composition: the March batch, with a lower fruit content and reduced starch proportion, resulted in greater rigidity. Conversely, the higher starch content of the October batch, due to increased fruit presence, contributed to greater flexibility but slightly reduced strength. These findings highlight the significant influence of raw material composition on the mechanical properties of chestnut-based biocomposites, emphasizing the need to balance starch and fiber ratios for specific applications.

Overall, the biocomposites produced in this study demonstrated lower mechanical properties compared to those of other chestnut-based composites reported in the literature. For example, chestnut bur composites using a polyester resin matrix have reported Flexural Strengths of 33.6 MPa [17], and chestnut shell composites with a polyester matrix reach up to 62.19 MPa [18]. Composites made with chestnut wood flour and a vinyl ester matrix show even higher Flexural Strength at 135 MPa [23]. Regarding the Flexural Modulus, chestnut shell composites with epoxy resin achieve up to 1736 MPa [21], while chestnut bur composites using similar starch-based compositions are around 4.85 MPa [16]. The lower mechanical values in this study can be attributed to the absence of synthetic matrices or additional reinforcements, as the composites were produced using only chestnut shells and kernel (starch) as the matrix, with fibers from inner shells as reinforcement. In contrast, studies utilizing synthetic or reinforced matrices typically report significantly enhanced mechanical properties. These findings align with the trends reported in the literature review [13], which highlight the lower mechanical performance of starch-based chestnut composites compared to that of synthetic matrix systems, reinforcing the trade-off between sustainability and mechanical strength.

### 3.4. Biocomposite Characterization

**Figure 4:**
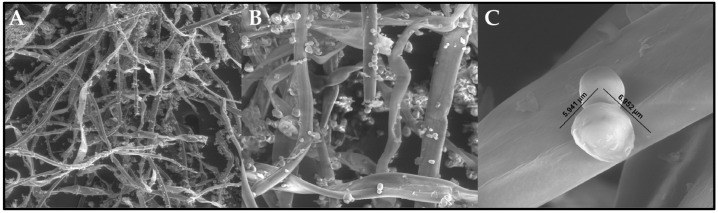
SEM micrographs of the optimized chestnut-based biocomposite (70% chestnut, 0% glycerol, processed at 120 °C) at different magnifications: (**A**) 400 µm, (**B**) 100 µm, and (**C**) 10 µm.

Scanning Electron Microscopy (SEM) was used to observe the morphology of the optimized ground biocomposite. Figure 4 shows the SEM micrographs of the composition of the ground optimized chestnut-based biocomposite (70% chestnut, 0% glycerol) at different magnifications as follows: 400 μm (Figure 4A), 100 μm (Figure 4B), and 10 μm (Figure 4C). As observed, the composition of the developed biocomposite includes fibers isolated from chestnut shells (~17 μm diameter) that act as a reinforcement agent of the polymeric matrix composed of starch granules with an average diameter of around 6 µm. The SEM images reveal a porosity attributed to the fibrous reinforcement and absence of synthetic binders, leading to incomplete densification. While glycerol could reduce porosity by improving matrix flowability, flexural tests showed superior performance in glycerol-free composites, making this formulation more advantageous. In the literature, bio-based composites show porosity levels ranging between 3.5 and 21% depending on the methodology as well [43].

#### 3.4.1. Wettability

**Figure 5:**
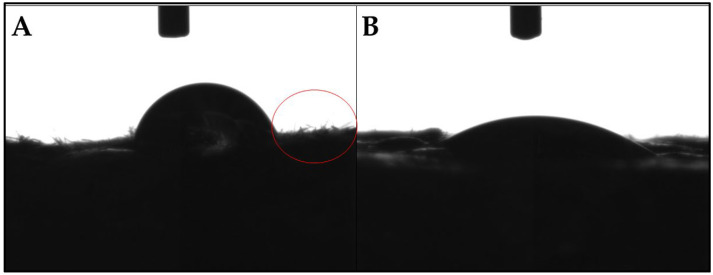
Water droplets on the surface of composites without (**A**) and with (**B**) shellac, where the red circle highlights vertically aligned fibers.

Wettability analysis indicates the hydrophobic or hydrophilic nature of a surface [44]. Higher contact angles signify greater hydrophobicity, while lower angles denote increased hydrophilicity. The results showed that the untreated composite had a higher contact angle (71.70 ± 14.21) (Figure 5A) compared to that of the composite treated with shellac (42.81 ± 5.79) (Figure 5B). This suggests that the shellac-treated surface was more hydrophilic, contrary to the initial expectations that shellac would enhance water resistance [26]. A potential explanation for this outcome is the effect of shellac on fiber orientation. Fibers stand more upright without shellac, creating a textured surface that may repel water more effectively. Fibers align more flatly with shellac, resulting in a smoother surface that reduces water repellence. Images from the contact angle measurements confirmed this difference, showing a more uniform and less textured surface in the shellac-treated composite. Despite these findings, both composite surfaces are classified as hydrophilic (10° < θ < 90°) [45]. For context, this study is the first to evaluate the wettability of chestnut-based biocomposites, making it a pioneering contribution in this area. No prior studies have reported contact angle measurements for similar systems, requiring comparisons with other bio-based and polymeric materials. Similar materials have shown contact angles within a comparable range, such as polyethylene-based composites, which showed contact angles around 62° [46], while cork was measured at 90° [47]. Although the shellac-treated composite demonstrated higher surface-level hydrophilicity, this does not necessarily translate to reduced water resistance overall. To fully understand the material’s performance, comprehensive water absorption tests involving prolonged submersion are needed to assess long-term water resistance.

#### 3.4.2. Water Absorption

Water absorption tests revealed that biocomposites without glycerol absorbed 10% water at 2 h, increasing to 45.2% at 24 h and reaching about 60% at 96 h due to the hydrophilic nature of starch and fibers [48,49]. Composites with glycerol showed even higher absorption, starting at 12.4% at 2 h, increasing to 52.3% at 24 h, and surpassing 65% by 96 h, attributed to glycerol’s moisture-attracting properties [50]. Shellac-treated composites demonstrated the lowest water absorption, with 3.6% at 2 h, 18.3% at 24 h, and approximately 30% at 96 h, showcasing shellac’s effective water resistance [26]. Although these composites exhibited higher surface wettability, they maintained superior long-term water resistance. For comparison, composites with 20% chestnut shell fibers and PHAs absorbed 15% over 60 days [19], while those with 40% chestnut shell fibers and polypropylene absorbed just 0.27% in one day [22]. This study demonstrates that chestnut-only and glycerol-containing composites absorbed over 60% of water in 4 days, highlighting the need for hydrophobic matrices to enhance water resistance.

#### 3.4.3. Thermal Conductivity

The chestnut-based composite (75% chestnut, 30% glycerol at 120 °C) exhibited a thermal conductivity of 0.79 W/m·K after 53 min at 48 °C, categorizing it as a thermal insulator. Since glycerol has a significantly lower thermal conductivity (0.28 W/m·K) [51], its presence likely contributes to the reduction in heat transfer, further enhancing the insulation properties of the composite. This value is in line with previous reports on chestnut shell fillers/epoxy composites, which exhibited a slightly lower thermal conductivity of 0.5 W/m·K [21]. The variation may be attributed to differences in matrix composition and the presence of natural voids, which influence heat transfer. This value is higher than cork (0.04 W/m·K) and oak wood (0.16 W/m·K) but comparable to brick (0.72 W/m·K) and much lower than glass (1.4 W/m·K) and metals like aluminum (177 W/m·K) [52]. The results indicate the composite’s suitability for insulation applications.

#### 3.4.4. TGA and DSC

**Figure 6:**
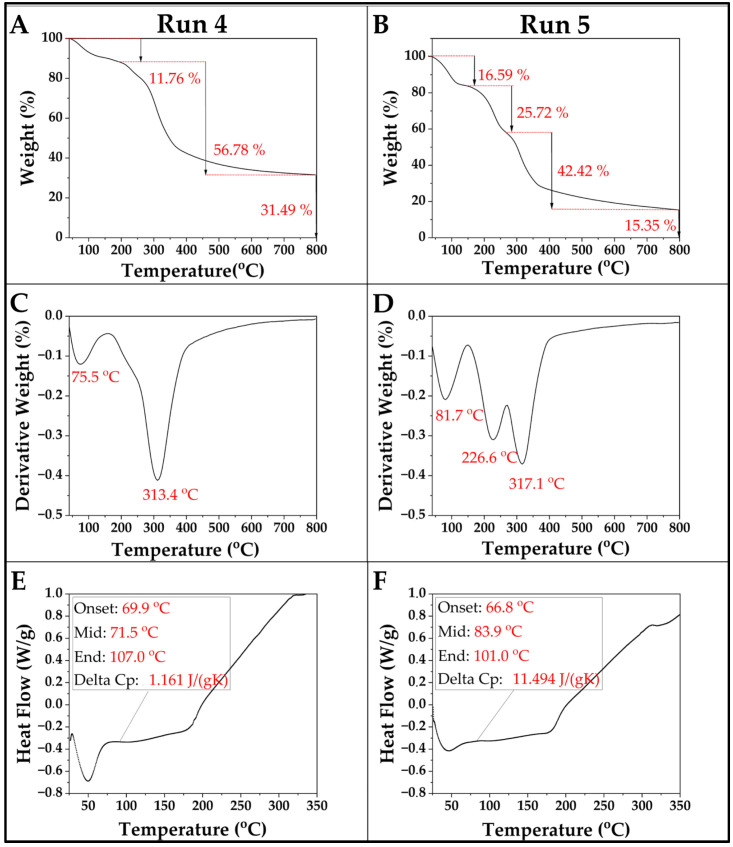
TGA curves for composites’ run 4 (without glycerol) (**A**) and run 5 (with glycerol) (**B**), and their corresponding DTG curves (**C**,**D**). DSC curves showing glass transition temperature (Tg) values for composites’ run 4 (**E**) and run 5 (**F**).

TGA showed distinct thermal behaviors for chestnut-based composites with varying glycerol content. In Figure 6A,B the mass losses are visible, while in Figure 6C,D, the temperatures at which these mass losses occur are shown. For run 4 (75% chestnut, 0% glycerol at 80 °C), degradation began at 75.5 °C due to moisture loss, with the main decomposition at 313.4 °C, involving the breakdown of hemicellulose, cellulose, and partial lignin [53]. Run 5 (75% chestnut, 30% glycerol at 80 °C) showed a similar initial moisture loss at 81.7 °C, with the main decomposition slightly shifted to 317.1 °C, indicating a minor stabilizing effect from glycerol. An intermediate stage of decomposition at 226.6 °C aligned with known glycerol breakdown temperatures [42]. Run 4 had a higher ash residue (31.49%) compared to that of run 5 (15.35%), suggesting glycerol facilitated more complete combustion. Overall, glycerol altered the thermal profile, implying interactions that influenced stability and degradation pathways. This differs from composites with synthetic matrices, like chestnut shell composites with kaolin/polyester, which exhibit higher primary decomposition temperatures (~420 °C) [18].

DSC provided insights into the thermal transitions of chestnut-based composites (Figure 6E). In run 4 (75% chestnut, 0% glycerol, processed at 80 °C), the glass transition (Tg) began at 69.9 °C, with a midpoint of 71.5 °C, and ended at 107.0 °C, indicating a rigid structure and limited molecular mobility, reflected in a low ΔCp of 1.161 J/(g·K). In contrast, run 5 (75% chestnut, 30% glycerol, processed at 80 °C) (Figure 6F) exhibited a lower Tg onset at 66.8 °C, with a midpoint at 83.9 °C and an end at 101.0 °C, alongside a higher ΔCp of 11.494 J/(g·K). This shift suggests that glycerol, acting as a plasticizer, increased chain mobility and flexibility, characteristic of starch-based materials [54]. The higher Tg of the composite in this study compared to in a similar formulation (Tg at 71.9 °C for 79% starch, 7.5% chestnut burr, 0.75% glycerol) and PHA/chestnut shell composites (Tg from −9.6 °C to +3.9 °C) [19] indicates greater thermal stability [16].

### 3.5. Application: Candle Holder

**Figure 7:**
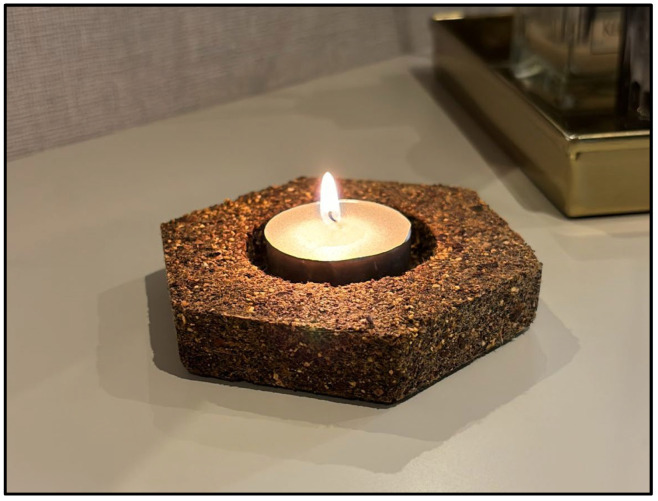
Prototype of a candle holder made from a chestnut-based biocomposite (75% chestnut, 0% glycerol, treated with shellac, processed at 120 °C).

A prototype candle holder was produced using a composite of 75% chestnut, 0% glycerol, treated with shellac, and processed at 120 °C (Figure 7). Although 70% chestnut was found to be optimal for mechanical properties, 75% was chosen to ensure compaction during molding. Glycerol was excluded to minimize water absorption and prevent mold growth, maintaining structural integrity. This pioneer prototype illustrates the practical application of this research findings, showcasing how chestnut waste can be transformed into a functional product, emphasizing sustainable practices in waste valorization.

## 4. Conclusions

This study highlighted the potential use of non-commercial chestnuts to produce biocomposites. The optimal composition for mechanical properties was identified as 70% chestnut, no glycerol, and processed at 120 °C (2.93 MPa for 30 min), yielding a Flexural Strength of 9.00 ± 0.40 MPa and a Flexural Modulus of 950 ± 11 MPa, closely matching the model predictions. Water interaction tests demonstrated that shellac-treated biocomposites reduced water absorption by approximately two times, enhancing moisture resistance, while glycerol increased water absorption due to its hygroscopic nature. Thermal analysis revealed that the composite without glycerol was more rigid while adding glycerol increased flexibility and slightly improved thermal stability. Additionally, the chestnut-based biocomposite exhibited a thermal conductivity of 0.79 W/m·K, classifying it as a thermal insulator, suitable for applications requiring insulation. A practical demonstration of the research involved successfully crafting a functional candle holder from chestnut-based biocomposites, showcasing the potential for sustainable waste utilization. This study underscores the viability of incorporating natural materials into functional products, offering an environmentally friendly alternative to fossil-based plastics and contributing to sustainability.

## Figures and Tables

**Table 1:** Experimental design for evaluation of the effects of chestnut (X1, %), glycerol (X2, %), and temperature (X3, °C) on Flexural Strength (Y1) and Flexural Modulus (Y2) of chestnut-based composites.

Run	Independent Variables			Dependent Variables			
	X1 ^a^ (%)	X2 (%)	X3 (°C)	Y1 (MPa)		Y2 (MPa)	
				Exp ^b^	Pred ^c^	Exp ^b^	Pred ^c^
1	65	15	120	5.9 ± 1.3	6.1	350 ± 120	343
2	65	30	100	0.55 ± 0.14	0.9	17.3 ± 3.8	−3
3	75	15	100	3.21 ± 0.51	3.9	160 ± 17	160
4	75	0	80	3.9 ± 1.7	4.7	390 ± 120	470
5	75	30	80	0.177 ± 0.020	−1.3	4.31 ± 0.65	3.8
6	85	15	120	4.17 ± 0.36	6.1	192 ± 17	225
7	75	0	120	9.90 ± 0.14	9.1	850 ± 280	910
8	65	0	100	8.7 ± 1.0	6.9	940 ± 230	840
9	75	15	100	4.3 ± 2.4	3.9	130 ± 20	160
10	65	15	80	0.44 ± 0.15	1.8	20 ± 11	90
11	75	30	120	3.6 ± 2.9	3.1	64 ± 18	54
12	85	30	100	1.04 ± 0.20	0.9	30.8 ± 5.3	61
13	85	15	80	1.40 ± 0.46	1.7	41 ± 11	−22
14	75	15	100	2.46 ± 0.39	3.9	110 ± 19	160
15	85	0	100	6.83 ± 0.78	6.9	590 ± 130	540
16	75	15	100	5.42 ± 0.53	3.9	270 ± 73	160
17	75	15	100	4.27 ± 0.17	3.9	180 ± 41	160

^a^ Reference to chestnuts unsuitable for sale. ^b^ Experimental values are expressed as mean ± standard deviation (n = 3). ^c^ Predicted values based on BBD evaluation.

## Data Availability

Data are contained within the article and Supplementary Materials.

## Supplementary Materials

The following supporting information can be downloaded at: https://www.mdpi.com/article/10.3390/polym17050616/s1, Figure S1: Press (A), mold (160 mm) (B) used to produce composites, and hydraulic press and aluminium mold (C) developed to produce a chestnut-based candle holder; Figure S2: FTIR spectra of shellac (SL) and non-commercial chestnut (NC); Figure S3: Actual values vs. Predicted values (based on BBD evaluation) for Flexural Strength (A) and Elastic Modulus (B) of biocomposites; Table S1: Actual levels at coded factor levels of independent variables used in the RSM.; Table S2. ANOVA and fit statistics.
