# Supplementary material for: Eco-Friendly Biocomposites from Chestnut Waste: Production, Optimization, Characterization, and Application
Source: Polymers (Basel). 2025 Feb 25;17(5):616. doi: 10.3390/polym17050616 (PMC11902815; doi:10.3390/polym17050616)
Supplement: Supplementary file 1 [file polymers-17-00616-s001.zip › polymers-3469149-supplementary.pdf]

Supplementary Materials

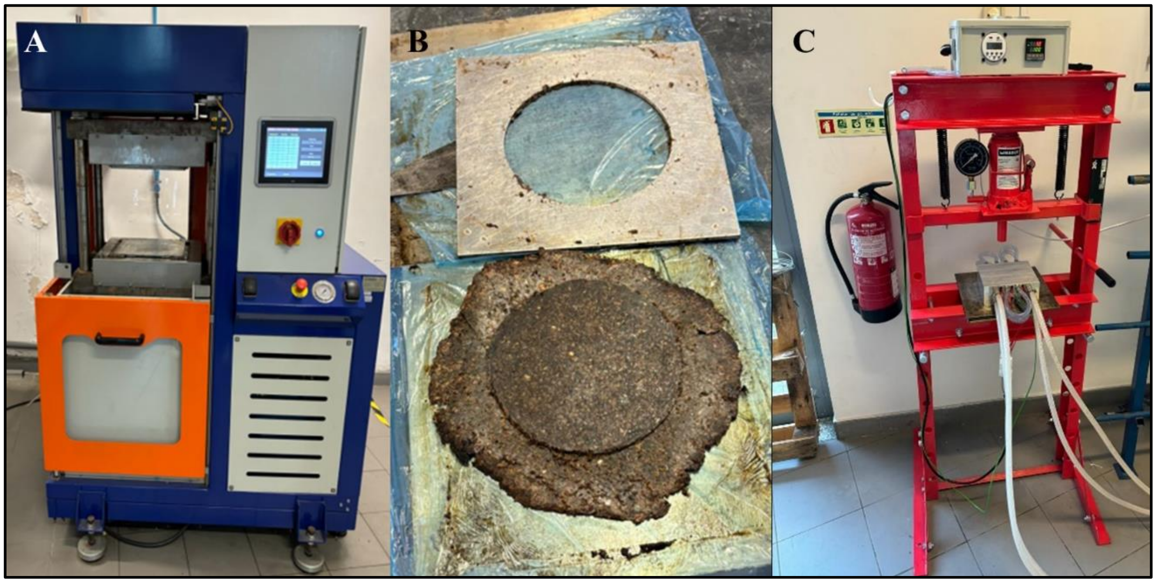

Figure S1. Press (A), mold (160 mm) (B) used to produce composites, and hydraulic press and aluminium mold (C) developed to produce a chestnut-based candle holder.

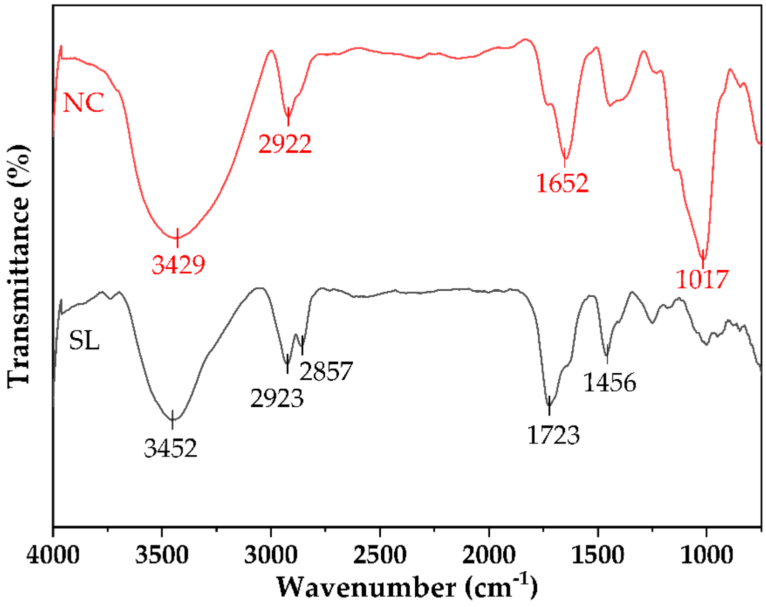

Figure S2. FTIR spectra of shellac (SL) and non-commercial chestnut (NC).

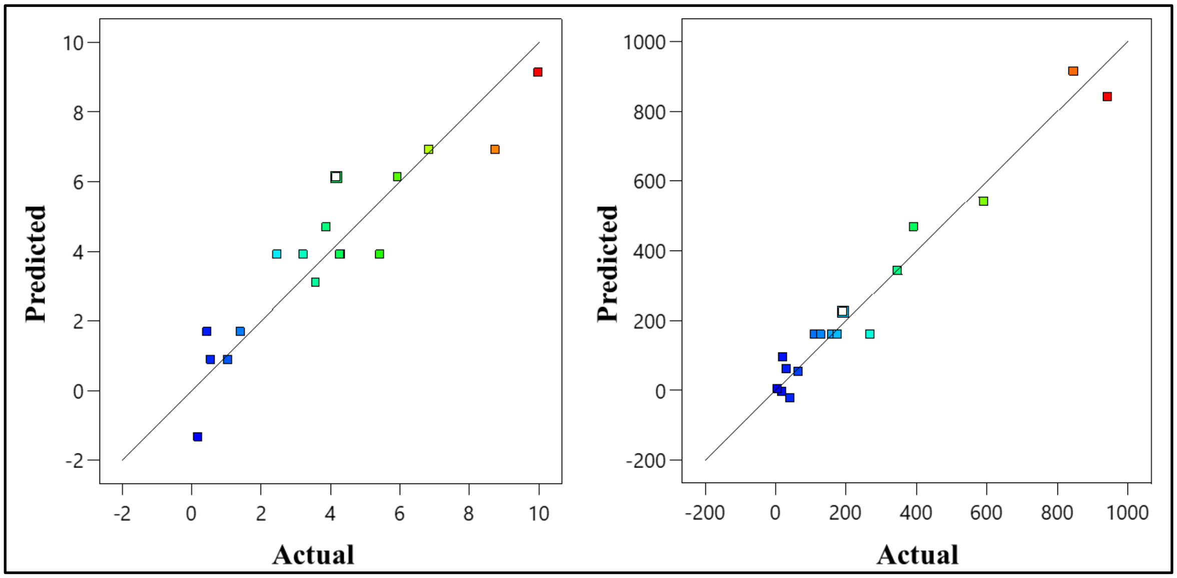

Figure S3. Actual values vs. Predicted values (based on BBD evaluation) for Flexural Strength (A) and Elastic Modulus (B) of biocomposites.

Table S1. Actual levels at coded factor levels of independent variables used in the RSM.

**Symbol**		**Low**	**Center**	**High**
		**Coded level**		
		**-1**	**0**	**1**
		**Actual level**		
X1	Chestnut (%)	65	75	85
X2	Glycerol (%)	0	15	30
X3	Temperature (°C)	80	100	120

Table S2. ANOVA and fit statistics.

Modified Linear Model for Flexural Strength				
**Source**	**Sum of Squares**	**Mean Square**	**F value**	***p* value**
Model	112.03	56.01	43.59	<0.0001*
X2-Glycerol	72.60	72.60	56.49	<0.0001*
X3-Temperature	39.43	39.43	30.68	<0.0001*
Residual	17.99	1.29		
Lack of Fit	12.84	1.28	1.00	0.55
Pure Error	5.15	1.29		
**Total**	130.02			
**Fit Statistics**				
R^2	0.8616			
Adjusted R^2	0.8419			
Predicted R^2	0.7894			
Adeq Precision	21.98			
**Modified Quadratic Model for Elastic Modulus**				
**Model**	1.27×10^6	2.12×10^5	41.47	< 0.0001*
X1-Chestnut	27976.77	27976.77	5.47	0.0414*
X2-Glycerol	8.79×10^5	8.79×10^5	171.91	< 0.0001*
X3-Temperature	1.23×10^5	1.23×10^5	24.05	0.0006*
X1X2	33432.29	33432.29	6.54	0.0285*
X2X3	38911.51	38911.51	7.61	0.0202*
$X2^2$	1.70×10^5	1.70×10^5	33.25	0.0002*
**Residual**	51127.14	5112.71		
Lack of Fit	35786.32	5964.39	1.56	0.3484
Pure Error	15340.82	3835.20		
**Total**	1.32×10^6			
**Fit Statistics**				
R^2	0.9614			
Adjusted R^2	0.9382			
Predicted R^2	0.8326			
Adeq Precision	20.43			

$*p < 0.05$.
